# Initiation of X Chromosome Inactivation during Bovine Embryo Development

**DOI:** 10.3390/cells9041016

**Published:** 2020-04-19

**Authors:** Bo Yu, Helena T. A. van Tol, Tom A.E. Stout, Bernard A. J. Roelen

**Affiliations:** 1 Farm Animal Health, Department of Population Health Sciences, Faculty of Veterinary Medicine, Utrecht University, 3584CM Utrecht, The Netherlands; B.yu@uu.nl (B.Y.); h.t.a.vantol@uu.nl (H.T.A.v.T.); 2 Equine Sciences, Department of Clinical Sciences, Faculty of Veterinary Medicine, Utrecht University, 3584CM Utrecht, The Netherlands; t.a.e.stout@uu.nl; 3 Embryology, Anatomy and Physiology, Department of Clinical Sciences, Faculty of Veterinary Medicine, Utrecht University, 3584CT Utrecht, The Netherlands

**Keywords:** XCI, bovine, embryo, *XIST*, H3K27me3, inner cell mass

## Abstract

X-chromosome inactivation (XCI) is a developmental process that aims to equalize the dosage of X-linked gene products between XY males and XX females in eutherian mammals. In female mouse embryos, paternal XCI is initiated at the 4-cell stage; however, the X chromosome is reactivated in the inner cell mass cells of blastocysts, and random XCI is subsequently initiated in epiblast cells. However, recent findings show that the patterns of XCI are not conserved among mammals. In this study, we used quantitative RT-PCR and RNA in situ hybridization combined with immunofluorescence to investigate the pattern of XCI during bovine embryo development. Expression of *XIST* (X-inactive specific transcript) RNA was significantly upregulated at the morula stage. For the first time, we demonstrate that *XIST* accumulation in bovine embryos starts in nuclei of female morulae, but its colocalization with histone H3 lysine 27 trimethylation was first detected in day 7 blastocysts. Both in the inner cell mass and in putative epiblast precursors, we observed a proportion of cells with *XIST* RNA and H3K27me3 colocalization. Surprisingly, the onset of XCI did not lead to a global downregulation of X-linked genes, even in day 9 blastocysts. Together, our findings confirm that diverse patterns of XCI initiation exist among developing mammalian embryos.

## 1. Introduction

In placental mammals, dosage compensation for X-encoded genes between female (XX) and male (XY) cells is achieved by inactivation of one of the two X chromosomes in female cells. This so-called X-chromosome inactivation (XCI) leads to stochastic transcriptional silencing of one of the two female X chromosomes, and is effected by the long non-coding RNA *XIST* [1,2]. *XIST* accumulates in “clouds” along the future inactive X chromosome (Xi), which can be visualized using RNA fluorescence in situ hybridization (FISH). To silence the chromatin and repress the X-linked genes from that chromosome, *XIST* recruits epigenetic modifiers [3]. Histone H3 lysine 27 trimethylation (H3K27me3) is a representative epigenetic hallmark associated with gene silencing and reported to be enriched on the Xi, and indeed colocalizes with *XIST* RNA [4].

X-chromosome inactivation is established during early embryonic development and maintained thereafter [5]. In the mouse, imprinted XCI is initiated at the 4-cell stage embryo stage with exclusive inactivation of the paternal X chromosome (Xp). Indeed, the paternal X chromosome remains inactive in trophectoderm (TE) cells during and after formation of the mouse blastocyst [6,7]. In the inner cell mass (ICM) of female embryos, however, the inactive Xp is reactivated, resulting in two active X chromosomes (XaXa). At around the time of implantation when the epiblast is being established, XCI is re-established with either maternal or paternal X chromosomes inactivated randomly in different cells, a process known as random X chromosome inactivation [8]. Although the initiation of XCI has been extensively explored in mouse embryos, the pattern of XCI in other mammalian species is less clear. In rabbits and humans for example, XCI starts later at the morula and blastocyst stages, respectively, and is not subject to imprinting [9]. Furthermore, large *XIST* clouds have been detected around both X chromosomes in cells of rabbit and human embryos, even in the ICM [9,10]. It appears, therefore, that XCI does not follow a uniform pattern in mammals.

In female mice, one of the defining characteristics of the naïve pluripotent state is the presence of two active X chromosomes; i.e., XaXa [11]. Similarly to the ICM, the absence of *XIST* expression from both X chromosomes has been observed in female mouse embryonic stem (ES) cells and induced pluripotent stem cells [12]. Moreover, the pluripotency factors OCT4, SOX2, and NANOG have been implicated in suppression of *XIST* expression, by binding to its first intron [13]. Upon pluripotent cell differentiation, the pluripotency factors are transcriptionally downregulated and *XIST* expression increases, leading to random XCI [14]. In human cells, however, the correlation between pluripotency state and XCI is less clear [14]. Even though the XaXa state has been reported in female human ES cells, these cell lines were shown to be very unstable during passages [15]. Meanwhile, inactive X chromosomes (Xi) were detected in other human ES and induced pluripotent stem cells, with *XIST* coating and accumulation of heterochromatin markers on the Xi [16]. Interestingly, female mouse epiblast stem cells, considered to be at a primed pluripotency state, also exhibit random XCI and share several morphological and molecular similarities with human ES cells. It has been hypothesized that human ES cells are at a primed pluripotent state, and that the presence of XaXa in female cells may still be a hallmark of naïve pluripotency in human cells [16,17,18]. So far, however, the connection between pluripotency and XCI state in other mammals has not been investigated in depth. Interestingly, stable primed pluripotent embryonic stem cell lines have recently been established from bovine embryos; however, their X chromosome activation states have not yet been reported [19].

One of the major consequences of XCI is the downregulation of X-linked gene expression on the inactivated X chromosome. During mouse development, silencing of X-linked genes follows the *XIST* coating [8,20]. Despite the *XIST* coating of the inactivated X chromosome, several X-linked genes escape silencing and are still expressed, as has been demonstrated in human and rabbit embryos [9]. In bovine blastocysts, X-linked genes were expressed at higher levels in females compared with males, suggesting that X-chromosome inactivation was not yet operational [21]. These data suggest that downregulation of X-linked genes after XCI initiation is not uniformly conserved among species.

Here, we examined the timing of XCI, its initiation in different lineage segregation, and regulation of X-linked gene expression during bovine embryo development. By performing quantitative RT-PCR, immunofluorescence, and RNA in situ hybridization, we established that *XIST* expression is initiated at around the morula stage and can be detected in both the ICM and TE, but does not lead to chromosome-wide downregulation of gene expression.

## 2. Materials and Methods

### 2.1. In Vitro Embryo Production and Collection of Samples

Bovine ovaries collected from a local slaughterhouse were transported to the laboratory in a thermos flask and rinsed with water at 30 °C. After rinsing, the ovaries were immersed in 0.9% NaCl supplemented with penicillin/streptomycin (PS) (100 µg/mL) at 30 °C. Cumulus-oocyte complexes (COCs) were aspirated from follicles with of diameter 2–8 mm using a winged infusion set (18G) connected to a vacuum aspiration system. Aspirated follicular fluid was collected in 50 mL centrifuge tubes. To isolate the COCs, sediment from the fluid was transferred to Petri dishes and COCs were identified using a stereomicroscope. The COCs were then matured by 23 h incubation at 39 °C in a humidified atmosphere of 5% CO_2_ in air, as described previously [22]. Next, the matured oocytes were fertilized with 1 × 10^6^/mL sperm cells. Presumptive female or male embryos were generated by using X-sorted or Y-sorted sperm (CRV, Arnhem, the Netherlands). Prior to introduction, sperm motility was checked under a stereomicroscope, and the moment that COCs were co-incubated with sperm was considered day 0. After incubation with sperm for 20–22 h, COCs were denuded by vortexing for 3 min. The presumptive zygotes were then placed in synthetic oviductal fluid (SOF) [23] for further development in a humidified atmosphere containing 5% CO_2_ and 7% O_2_ at 39 °C. At day 5, developing embryos were transferred to fresh SOF and further cultured until day 9.

For subsequent analysis, germinal vesicle (GV) oocytes and metaphase II (MII) oocytes were collected immediately after COC recovery and after 23 h in vitro maturation culture, respectively. Zygotes, and 2, 4, and 8 cell embryos were collected at 20, 32, 38 and 56 h after the start of fertilization respectively, and morulae and blastocysts were collected after 5 and 8 days, respectively of in vitro culture.

Inner cell mass (ICM) and trophectoderm (TE) cells were separated and collected from day 9 blastocysts using tungsten needles, as described previously [22]. Isolated ICMs and TE cells were immediately placed in groups (range 43–59) in RNase free tubes on ice containing 100 µL RLT buffer (Qiagen, Venlo, The Netherlands) and then stored at −80 °C until RNA isolation.

### 2.2. Isolation of Oviduct Cells and Cell Culture

Oviducts were collected from a local slaughterhouse and transported to the laboratory on ice. After removal of surrounding tissue, the oviducts were washed three times in PBS supplemented with penicillin (10 µg/mL) and streptomycin (10 µg/mL) (Gibco). Oviduct luminal epithelial cells were collected from the ampullary end of the oviducts by squeezing. The cells were washed twice in HEPES-buffered Medium 199 (Gibco) supplemented with penicillin (100 µg/mL), streptomycin (100 µg/mL), and gentamicin (50 µg/mL, Sigma, G1272) and centrifuged at 500× *g* for 5 min at 25 °C. The cells were then cultured for 24 h at 37 °C with 5% CO_2_ in HEPES-buffered Medium 199 supplemented with penicillin (100 µg/mL), streptomycin (100 µg/mL), gentamicin (50 µg/mL), and 10% fetal calf serum (FCS; Bovogen Biologicals, Melbourne, Australia).

HEK293 (human embryonic kidney) cells were grown in DMEM/F12 supplemented with penicillin (100 µg/mL), streptomycin (100 µg/mL), gentamicin (50 µg/mL), and 10% FCS at 37 °C with 5% CO_2_.

### 2.3. RNA Extraction and cDNA Generation

Oocytes and embryos in pools of 20 were washed in PBS and stored in RLT buffer at −80 °C until RNA extraction. Total RNA isolation was performed using the RNeasy Micro Kit (Qiagen, Valencia, CA, USA) according to the manufacturer’s instructions. Complementary DNA (cDNA) synthesis was carried out directly after RNA isolation. The reverse transcription (RT) mixture was prepared from 10 µL of the RNA sample, 4 µL of 5 × RT buffer (Invitrogen, Breda, The Netherlands), 10 mM DTT (Invitrogen), 0.5 mM dNTP (Promega), 8 units of RNAsin/ RNAse inhibitor (Promega), and 150 units Superscript III reverse transcriptase (Invitrogen) in a total volume of 20 µL. Minus-RT controls were made up of the same reagents as above, except the reverse transcriptase. The mixtures were incubated at 70 °C for 5 min, followed by 1 h at 50 °C, and 5 min at 80 °C. Samples were subsequently stored at −20 °C.

### 2.4. Quantitative Reverse Transcription-PCR

Primer pairs (Eurogentec) were designed on Primer-Blast (http://www.ncbi.nlm.nih.gov/tools/primer-blast) using *Bos taurus* mRNA (Genbank; http://www.ncbi.nlm.nih.gov/nucleotide) as the template. Amplification took place in 10 µL iQ SYBR Green supermix (Biorad), 9 µL RNAse and DNAse-free water (Invitrogen) and 1 µL cDNA with a final primer concentration of 500 nM. Reactions were performed in a CFX detection system (Biorad) following the manufacturer’s protocol. The mixture was kept at 95 °C for 3 min, followed by 40 cycles of 95 °C for 20 sec, the primer specific annealing temperature (Table S1) for 20 sec and extension at 72 °C for 20 sec. To verify the purity of the PCR products, melting curves were generated with temperature increments of 0.5 °C from 65 °C to 95 °C for 5 sec each step. All the reactions were performed on three independent cDNA samples in duplicate. To determine quantitative (q)RT-PCR amplification efficiency, standard curves for primers were made by 4-fold dilutions of cDNA from 400 oocytes. *YWHAZ*, *GAPDH,* and *SDHA* were used for normalization [24].

### 2.5. Combined RNA FISH and Immunofluorescence

Bovine *XIST* oligo probes (Table S2) were designed using the Stellaris online probe designer (https://www.biosearchtech.com/support/tools/design-software/stellaris-probe-designer) and *Bos taurus XIST* transcript variants X1, X2, and X3 (Genbank; http://www.ncbi.nlm.nih.gov/nucleotide) as the templates. Embryos and cells were fixed with 4% paraformaldehyde for 10 min. After washing twice in PBST (1x PBS, 0.01% Triton X-100), samples were permeabilized using Cytoskeletal Buffer (10 mM PIPES, pH 6.8; 100 mM NaCl; 300 mM sucrose; 3 mM MgCl_2_) containing 0.5% Triton X-100 on ice for 5 min. After three further washes in PBST, samples were kept in 70% ethanol at 4 °C for at least 2 h until further use.

After fixation and permeabilization, embryos and cells were washed twice in PBST and then incubated in dilution buffer (1x PBS, 0.3% Triton X-100, 0.4 units/mL RNAsin/ RNAse inhibitor) with the primary antibodies at 37 °C for at least 2 h. The primary antibodies were rabbit monoclonal antibody against H3K27me3 (Cell Signaling Technologies; #9733; 1:500), mouse monoclonal antibody against CDX2 (Biogenex; CDX2-88; 1:200), and mouse monoclonal antibody against NANOG (eBiosciences; 14-5768-82;1:250). Following three washes in PBST, samples were incubated with the secondary antibodies: goat anti mouse Alexa488 or goat anti rabbit Alexa568 (Invitrogen, Venlo, The Netherlands) at 37 °C for 1 h.

The RNA FISH protocol was based on the Stellaris protocol for adherent cells (https://biosearchassets.blob.core.windows.net/assets/bti_stellaris_protocol_adherent_cell.pdf) with a few modifications. Embryos or cells were incubated in Stellaris RNA FISH Buffer A (SMF-WA1-60, Biosearch Technologies) at 37 °C for 1 h and then in 250 nM Stellaris Probe (https://www.biosearchtech.com/products/rna-fish/custom-stellaris-probe-sets) diluted in Stellaris RNA FISH Hybridization Buffer (SMF-HB1-10, Bio-search Technologies) at 37 °C overnight. After hybridization, samples were incubated in Stellaris RNA FISH Buffer A at 37 °C for 30 min, followed by nuclear staining using DAPI (Sigma Aldrich) diluted in Buffer A. After three washes in RNA FISH Wash Buffer B (SMF-WB1-20, Bio-search Technologies), samples were mounted with Vectashield (Brunschwig Chemie, Amsterdam, The Netherlands) on Cavity slides with epoxy coating (VWR, 631-0457) and stored at 4 °C before imaging. Images were obtained using a confocal laser scanning microscope (SPE-II-DMI4000; Leica, Son, The Netherlands) and were further analyzed using Fiji (http://fiji.sc/Fiji) and IMARIS software (Bitplane, Zürich, Switzerland).

### 2.6. Gender Determination of Single Embryos

Individual embryos were collected from Cavity slides after imaging. To extract genomic DNA (gDNA) from single embryos, the prepGem kit (ZyGem, Hamilton, New Zealand) was used according to the manufacturer’s instructions. A 20 µL PCR mixture was made up of 10 µL iQ SYBR Green supermix (Biorad), 5 µL RNAse and DNAse-free water (Invitrogen), and 5 µL gDNA with final primer concentrations of 500 nM. The reaction steps were as described above for qRT-PCR. Presence of gDNA was confirmed using primers for Gremlin that were not separated by an intron (forward, 5′-CATCAACCGCTTCTGCTACG-3′; reverse, 5′-TGGCTGGAGTTCAGGACAGT-3′) and identification of embryos as male was determined using *SRY* (forward, 5′-ACAGTCATAGCGCAAATGATCAGTG-3′; reverse, 5′-GGGTTGCATAGTATTGAAGAGTCTGC-3′). Cycle threshold (Ct) values lower than 33 with appropriate melting curves were regarded as valid for gender determination.

### 2.7. Statistical Analysis

All calculations were carried out in Excel and statistical differences were examined using GraphPad Prism 7 (https://www.graphpad.com/scientific-software/prism/). Differences between two groups were determined by two-tailed unpaired Students *t*-tests, and differences between multiple groups were analyzed by one-way ANOVA, followed by a post-hoc Tukey test. Statistical significance was set at *p* < 0.05.

## 3. Results

### 3.1. Expression of Genes Related to X-Chromosome Inactivation

**Figure 1:**
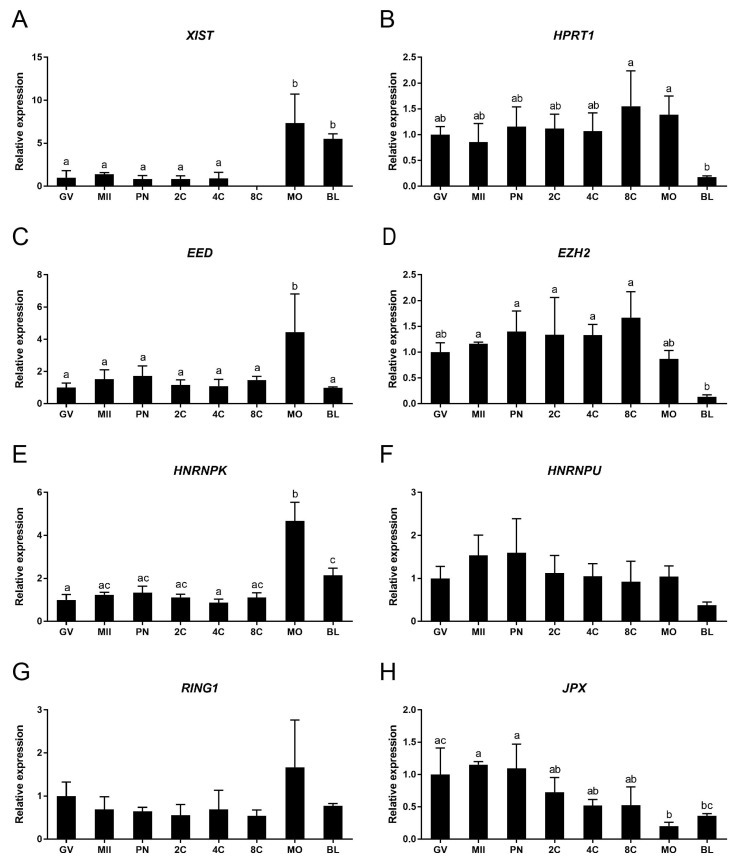
The relative expression of XCI related genes during *in vitro* bovine embryo development, as determined by quantitative RT-PCR. (**A**) *XIST*, (**B**) *HPRT1*, (**C**) *EED*, (**D**) *EZH2*, (**E**) *HNRNPK*, (**F**) *HNRNPU*, (**G**) *RING1*, (**H**) *JPX*. GV, MII, PN, 2C, 4C, 8C, MO, and BL refer to germinal vesicle, metaphase II, pronuclear, 2-cell, 4-cell, 8-cell, morula, and blastocyst stages, respectively. Embryos were derived by fertilization with non-sexed sperm. Relative expression in GV oocytes set at 1. Significant differences between bars are indicated by different letters above bars (*p* < 0.05). Error bars indicate standard deviations of three independent biological replicates.

X-chromosome inactivation is a dynamic process, and the timing of its initiation during preimplantation development varies among mammalian species [9]. Here we performed qRT-PCR to examine the relative expression of XCI related genes from bovine GV and MII stage oocytes and embryo stages up to day 8 blastocysts. The expression level of *XIST* was relatively low and stable at developmental stages ranging from the GV oocyte to the 4-cell embryo. Expression at the 8-cell embryo stage was below the detectable level, presumably due to degradation of maternal mRNA after embryonic genome activation. Expression of *XIST* increased significantly at the morula and blastocyst stages (Figure 1A). *HPRT1* is an X-linked gene known to be inactivated after XCI; its downregulation has been proposed as a marker for XCI [25,26]. Expression of *HPRT1* was detected in oocytes and embryo stages up to the blastocyst, but was significantly downregulated between the morula and the blastocyst stages (Figure 1B). Together, these data suggest that XCI expression starts around the morula stage in bovine embryos.

We also investigated gene expression for two members of the polycomb repressive complex 2 (PRC2); namely, EED and EZH2. PRC2 has been reported to accumulate on the inactive X chromosome and to be required for establishing histone methylation [27,28]. Expression of *EED* was relatively constant throughout embryonic development, but was significantly elevated at the morula stage (Figure 1C). *EZH2* expression was stable until the 8-cell stage, after which expression decreased gradually (Figure 1D).

HNRNPK can bind to *XIST* to recruit polycomb repressive complex 1 (PRC1) [29]. HNRNPU, another *XIST* RNA binding protein, was previously reported to be essential for *XIST* recruitment to the inactive X chromosome (Xi) [30]. *HNRNPK* (Figure 1E) exhibited a similar expression pattern to *EED*. The expression of *HNRNPU* was relatively constant throughout embryo development (Figure 1F).

We also examined *RING1* and *JPX* expression during embryo development. RING1, a polycomb repressive complex 1 factor, is involved in Ubiquitination of Histone H2A of Xi [31]. The expression of *RING1* was relatively stable in all oocyte and embryo groups (Figure 1G). *JPX*, a long noncoding X-linked RNA, has been reported to act as a molecular switch for XCI [32]. Surprisingly, we found expression of *JPX* to decrease at the morula stage (Figure 1H)—oppositely to the expression of *XIST*—suggesting that its function is not conserved between mouse and cow. We also examined expression of another major *XIST* activator, RNF12 [33]. Expression of *RNF12* was below the detectable level throughout embryo development.

### 3.2. Gene Expression Differences Between Male and Female Embryos

**Figure 2:**
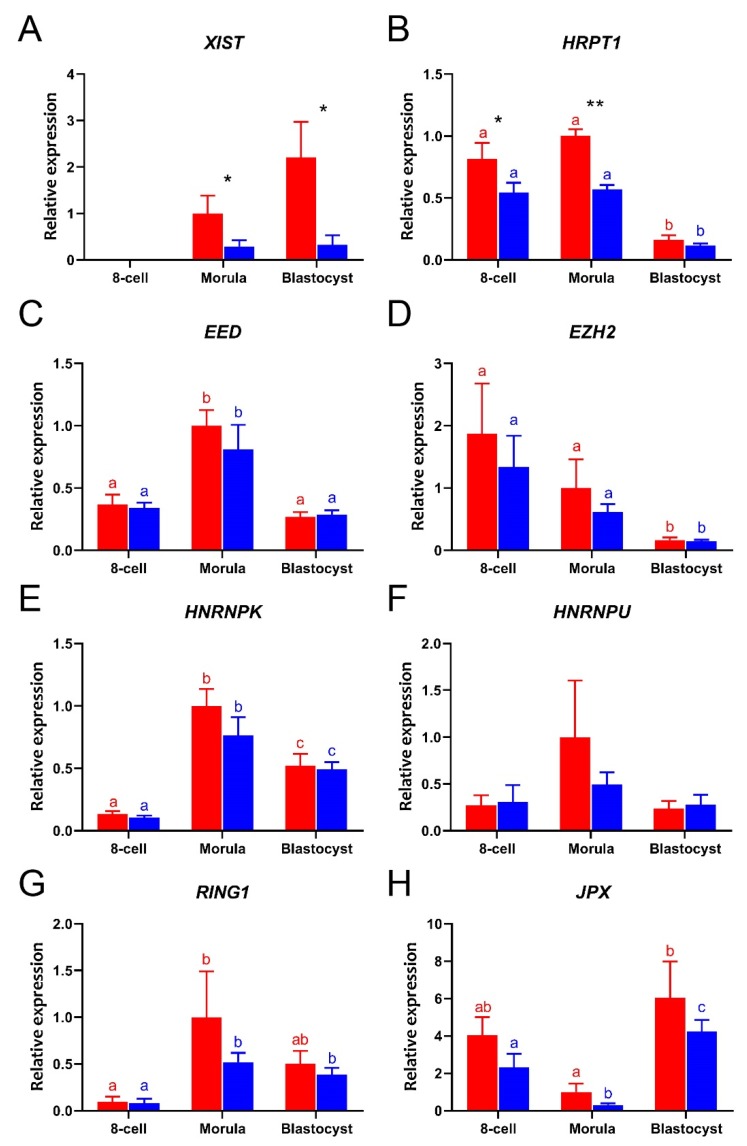
The relative expressions of XCI related genes in female (red bars) and male (blue bars) bovine embryos from 8-cell stage to day 8 blastocysts, as determined by quantitative RT-PCR. (**A**) *XIST*, (**B**) *HPRT1*, (**C**) *EED*, (**D**) *EZH2*, (**E**) *HNRNPK*, (**F**) *HNRNPU*, (**G**) *RING1*, (**H**) *JPX.* Relative expression from female morulae set at 1; * (*p* < 0.05) and ** (*p* < 0.005) indicate significant differences between females and males. Significant differences among embryos with same gender are indicated by different letters with the same color (*p* < 0.05). Error bars indicate standard deviations of three independent biological replicates.

To determine the differences in expression of XCI related genes in female and male embryos, we used sex-sorted sperm to fertilize oocytes. To check the accuracy of sperm selection, the sexes of single blastocysts made using X-sorted or Y-sorted sperm were determined by the PCR for *DDX3Y, USP9Y,* and *ZRSR2Y* on genomic DNA from blastocysts. Control experiments indicated that sorted sperm was indeed suitable for generation of sex-specific embryos (Table S3) [34]. Here, we focused on the period between the 8-cell stage and the blastocyst, since we found most changes in XCI related gene expression to arise after the 8-cell stage (Figure 1). Expression of all the selected XCI-related genes was detected in both female and male embryos, with the exception of *XIST* at the 8-cell stage (Figure 2A). The expression of *HPRT1* was significantly higher in female than male embryos (Figure 2B). As expected, significantly higher expressions of *XIST* and *HPRT1* were detected in female than in male morulae (Figure 2A,B). At the blastocyst stage, only *XIST* was expressed at higher levels in female than in male embryos (Figure 2C), while expression of the other XCI related genes showed no significant sex-related differences from the 8-cell stage embryo to the blastocyst (Figure 2C–H).

### 3.3. XIST RNA Detection in Female Morulae

**Figure 3:**
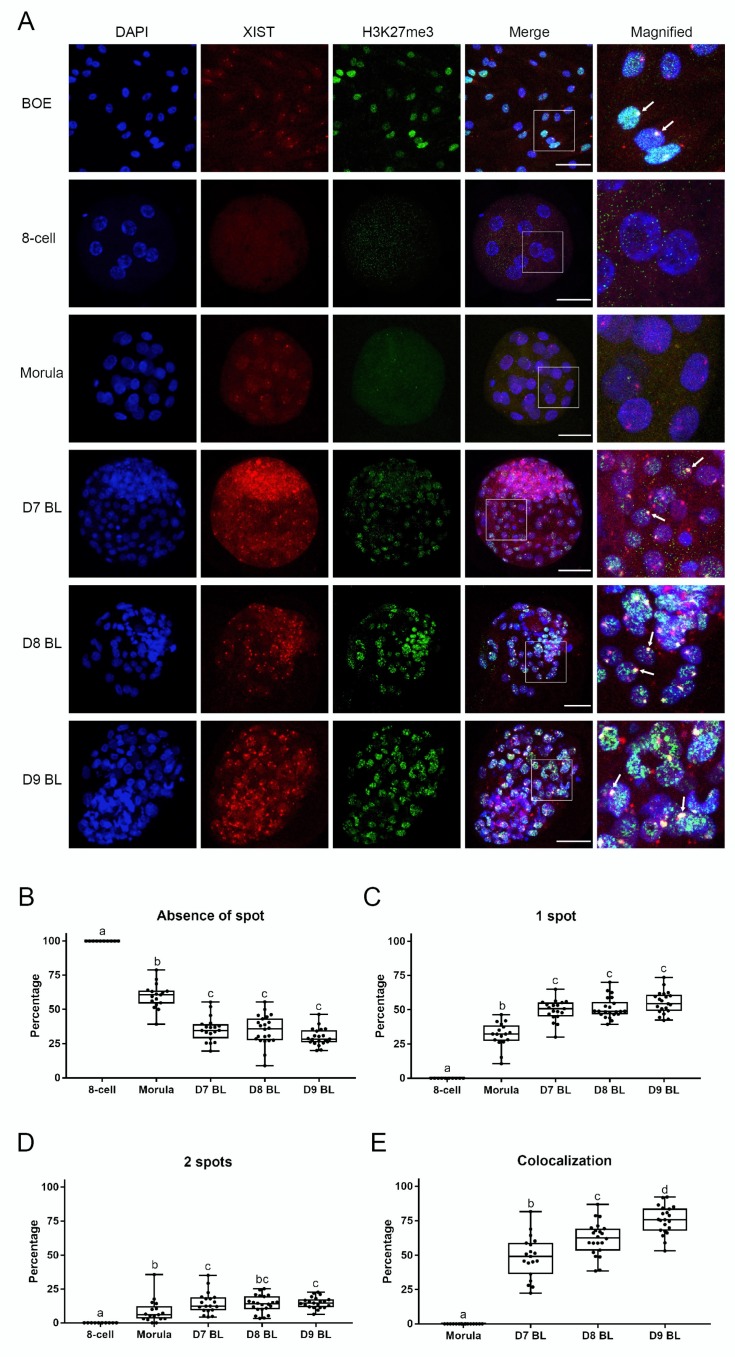
Combined XIST RNA FISH and H3K27me3 immunofluorescence in bovine oviduct epithelial cells and female embryos. XIST RNA FISH combined with H3K27me3 immunofluorescence in oviduct cells and the 8-cell embryos up to day (D) 9 blastocysts (**A**). XIST and H3K27me3 colocalization are indicated (arrow), scale bar = 50 µm. White boxes indicate areas presented in the right column at higher magnification. Percentages of cells lacking a XIST spot (**B**), 1 XIST spot (**C**), 2 XIST spots (**D**) from 8-cell embryos up to D9 blastocysts. Percentages of XIST and H3K27me3 colocalization from morula to day 9 blastocyst (**E**). Significant differences between boxes are indicated by different letters (*p* < 0.05). BL = blastocyst.

One of the hallmarks of XCI is *XIST* RNA accumulation on the Xi and colocalization with an area of extensive histone H3 lysine 27 trimethylation. The presence of *XIST* RNA can be detected by fluorescence in situ hybridization (FISH), while H3K27me3 can be detected by immunostaining. In both cases the Xi can be observed as a spot within the nucleus. To establish *XIST* RNA FISH combined with H3K27me3 immunofluorescence, we first optimized human *XIST* RNA FISH on HEK293 cells. These female cells are known for their abnormal chromosome numbers, including supernumerary X chromosomes. Indeed, using the RNA FISH method we detected multiple *XIST* spots in the nuclei of HEK293 cells (Figure S1A) [35]. Next, we tested the newly designed bovine *XIST* probe on bovine oviduct epithelial cells, and then combined staining with immunofluorescence for H3K27me3. As expected, one spot of *XIST* RNA was detected within the nuclei of most cells and colocalized with a H3K27me3 positive spot (Figure 3A).

We then addressed localization of *XIST* RNA and H3K27me3 in embryos from the 8-cell stage up to day 9 blastocysts. The sexes of individual embryos were determined by PCR, with the exception of 8-cell stage embryos, for which insufficient genomic DNA was available. In agreement to what we found by qRT-PCR (Figure 1A and Figure 2A), there was no expression of *XIST* in any 8-cell stage embryo we examined by RNA FISH; instead, spots of *XIST* were first detected in female embryos at the morula stage (Figure 3A, Figure S1B).

In fact, we observed three different patterns of *XIST* staining (no spot, one spot, or two spots) in embryonic nuclei. Nuclei without any *XIST* spots were found at all stages from the 8-cell stage embryo up to the day 9 female blastocyst. However, the percentage of nuclei without a *XIST* spot decreased markedly from 100% at the 8-cell stage to 60% in the morulae, and 36% in day 7 blastocysts (Figure 3B). Conversely, the percentage of nuclei with one *XIST* spot increased significantly from 0% at the 8-cell stage to 32% at the morula stage and 50% in day 7 blastocysts (Figure 3C). Nuclei with two *XIST* spots represented only small proportions of the total nuclei; namely, 9% at the morula stage and 15% in day 7 blastocysts (Figure 3D). Interestingly, the percentages of nuclei displaying the three different numbers of *XIST* spots were relatively stable across day 7 to day 9 blastocysts, indicating that the accumulation of *XIST* RNA was already established at day 7.

We then focused on the colocalization H3K27me3 spots with *XIST* RNA spots during embryo development. Very weak H3K27me3 staining was first detected at the morula stage; however, convincing H3K27me3 spots were absent at this stage. Clear H3K27me3 spots started to form in day 7 blastocysts, i.e., later than *XIST* RNA accumulation began, but they did consistently colocalize with the *XIST* RNA spots (Figure 3A). The percentage of *XIST* RNA and H3K27me3 spot colocalization increased from 0% in morulae to 49% in day 7 blastocysts, 62% in day 8 blastocysts, and 76% in day 9 blastocysts (Figure 3E). In short, H3K27me3 on the X chromosome started after *XIST* accumulation. In contrast, we did not find any *XIST* RNA or H3K27me3 spots in male embryos (Figure S1C). Based on these data, we conclude that XCI is initiated at the morula stage of bovine female embryo development.

### 3.4. Fewer XIST RNA Spots Were Present in the Inner Cell Mass than in the Trophectoderm

In female mouse blastocysts, *XIST* expression was low and no clear *XIST* spots were found in ICM cells [8]. In bovine embryos, however, we observed several ICM cells with distinct *XIST* RNA spots (Figure 3A). This suggests that XCI was already initiated in ICM cells during preimplantation development in bovine embryos.

**Figure 4:**
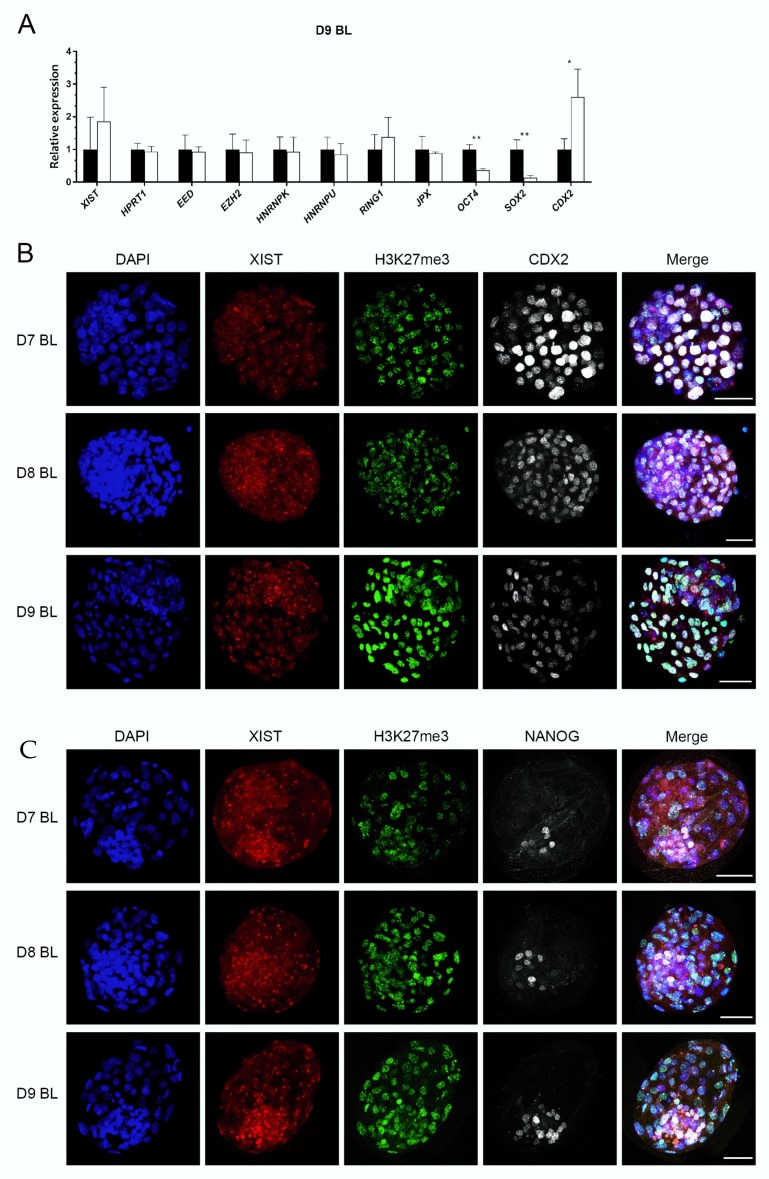
The relative expression of XCI related genes in inner cell mass (ICM) cells (black bars) and trophectoderm (TE) cells (white bars), and *XIST* RNA FISH combined with immunofluorescence in female embryos. The relative expression of XCI related genes (**A**). Relative expression in ICM set at 1; * (*p* < 0.05) and ** (*p* < 0.05) indicate significant differences. Error bars indicate standard deviations of three biological replicates. *XIST* RNA FISH combined with double staining of H3K27me3 and CDX2 (**B**) or NANOG (**C**) from day 7 (D7) to day 9 (D9) blastocysts, scale bar = 50 µm. BL = blastocyst.

To confirm *XIST* expression in ICM cells of the ICM, we examined ICM and TE cells separated mechanically. This was performed on embryos produced with unsorted sperm, since X and Y sorted sperm yield far fewer embryos, whereas we needed large numbers of blastocysts to obtain sufficient RNA from embryo fragments. The specificity of isolated ICM and TE fragments was determined by relative expression of *OCT4*, *SOX2,* and *CDX2*, with similar results to those reported previously [22] (Figure 4A). Interestingly, expression of *XIST* in ICM cells was at a higher level in TE cells, but this difference did not reach statistical significance (*p* > 0.361). In addition, other XCI related genes were expressed at similar levels in the ICM and TE (Figure 4A).

We then examined *XIST* RNA spots and their colocalization with H3K27me3 in ICM cells. TE cells were identified by CDX2 expression, and CDX2 negative cells were regarded as ICM. While putative epiblast precursors were stained with a NANOG antibody, NANOG negative cells were regarded as a combination of TE and hypoblast. Again, we detected nuclei with clear *XIST* spots in both CDX2 negative cells and NANOG positive cells; this included cells with colocalized *XIST* and H3K27me3 spots (Figure 4B,C). These data indicate that XCI in the blastocyst was already initiated in some ICM cells, including potential epiblast precursors.

**Figure 5:**
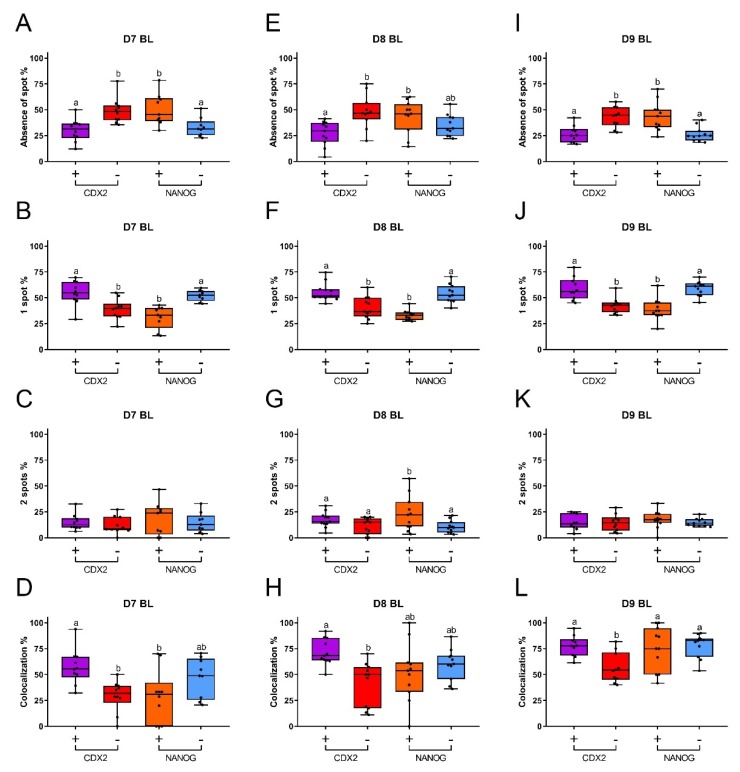
Percentages of nuclei with different numbers of *XIST* spots in CDX2 and NANOG positive or negative cells, and percentages of colocalization of *XIST* and H3K27me3 from day 7 to day 9 female blastocysts. Absence of an *XIST* spot (**A**), one *XIST* spot (**B**), two *XIST* spots (**C**), and colocalization of *XIST* and H3K27me3 (**D**) in day 7 (D7) blastocysts; absence of an *XIST* spot (**E**), one *XIST* spot (**F**), two *XIST* spots (**G**), and colocalization of *XIST* and H3K27me3 (**H**) in day 8 (D8) blastocysts; absence of an *XIST* spot (**I**), one *XIST* spot (**J**), two *XIST* spots (**K**), and colocalization of *XIST* and H3K27me3 (**L**) in day 9 (D9) blastocysts. Presence (purple box) and absence (red box) of CDX2 are indicated by + and -, respectively. Presence (orange box) and absence (blue box) of NANOG are indicated by + and -, respectively. Significant differences between boxes are indicated by different letters (*p* < 0.05). BL = blastocyst.

In order to understand the relationship between XCI and pluripotency in bovine embryos, we determined the percentages of nuclei with different numbers of *XIST* spots (no spot, one spot, and two spots) and their colocalization with H3K27me3. In day 7 blastocysts, the percentages of nuclei without *XIST* spots were similar in CDX2 negative and NANOG positive cells. The percentages of nuclei with no *XIST* spot in CDX2 positive and NANOG negative cells were also similar and were less abundant than among the CDX2 negative cells and NANOG positive cells (Figure 5A). More nuclei with a single *XIST* spot were detected in CDX2 positive and NANOG negative cells, compared to the potentially pluripotent CDX2 negative and NANOG positive cells (Figure 5B). Nuclei with two *XIST* spots were less common (less than 19%), and there was no significant difference between CDX2 positive, CDX2 negative, NANOG positive, and NANOG negative cells (Figure 5C). Day 8 and day 9 blastocysts showed similar patterns of *XIST* spot numbers to day 7 blastocysts, although a higher proportion of nuclei with two spots was found in NANOG positive cells of day 8 blastocysts (23%) (Figure 5E to Figure 5G and Figure 5I to Figure 5K). We also analyzed the frequency of *XIST* and H3K27me3 co-localization. More cells with colocalized *XIST* and H3K27me3 staining were observed in CDX2 positive than CDX2 negative cells, in blastocysts at all stages (day 7 to 9: Figure 5D,H,L). However, the frequency of co-localization was similar in NANOG positive and NANOG negative cells at all stages of blastocyst development, albeit that there was considerable variability between samples (Figure 5D,H,L). Combined, these data suggest that cells with a higher degree of pluripotency have a lower tendency to initiate XCI during embryo development.

### 3.5. X-Linked Gene Regulation after XCI Initiation

**Figure 6:**
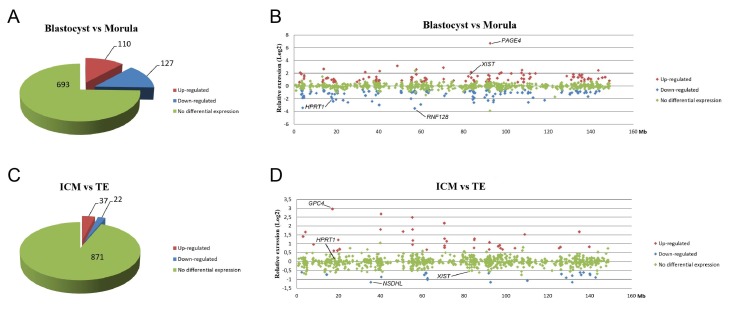
Proportional distribution of X-linked gene expression in blastocysts versus morulae and inner cell mass cells versus trophectoderm cells. Proportional distribution of upregulated (red), downregulated (blue), and equally expressed (green) X-linked genes in blastocysts versus morulae (**A**) and inner cell mass cells versus trophectoderm cells (**C**). The relative expression (Log2) of X-linked genes in blastocysts versus morulae (**B**) and inner cell mass cells versus trophectoderm cells (**D**) are plotted according to their locations on the X chromosome.

To assess whether the initiation of XCI triggered downregulation of X-linked gene expression during development, we analyzed previously generated microarray data in which we had compared gene expression levels between morulae and blastocysts [22]. In total, expressions of 930 genes located on the X chromosome were evaluated, with differential expression determined using cut-offs of 1.5-fold and *p* < 0.05 (Tables S4 and S5). The observation of a higher percentage of cells with *XIST* spots in blastocysts than morulae (Figure 3C,D), indicated that more cells start to undergo XCI during this transition. Despite an increase in *XIST* coating as observed as XIST spots, the increase was apparently not abundant enough in all cells to cause a significant change in X-linked gene expression. Indeed, most (693) X-linked genes showed no difference in expression and the number of downregulated genes in blastocysts compared to morulae (127) was similar to the number of genes that was upregulated (110) (Figure 6A). Nevertheless, a higher expression of *XIST* and lower expression of *HPRT1* in blastocysts was found in the microarray data (Figure 6B). We then compared the expression of X-linked genes in ICM cells and TE cells of day 9 blastocysts. Only a small number of X-linked genes was differentially expressed, of which 37 genes were upregulated and 22 genes were downregulated in ICM cells compared to TE cells (Figure 6C). Moreover, there was no significant difference in expression of *XIST* and *HPRT1* between ICM and TE cells (Figure 6D), which was consistent with our qRT-PCR findings (Figure 4A). Overall, the data suggest that in bovine embryos, XCI initiation does not lead to the downregulation of most X-linked genes.

## 4. Discussion

X chromosome dosage compensation between XX female and XY male cells is achieved by XCI during early female embryonic development. Although this process has been intensively studied in the mouse, recent studies showed different patterns of XCI in other mammals [9,36,37]. However, little is known about the timing of XCI initiation and its relationship with lineage segregation in non-rodent species. Therefore, we studied XCI in early bovine embryos.

*XIST*, one of the major effectors of XCI, accumulates on the future Xi and initiates silencing in *cis* [38]. In bovine sperm cells, the *XIST* gene does not appear methylated, suggesting that *XIST* could be expressed in these cells [39]. The absence of *XIST* transcripts at the 2-cell stage indicates that possible *XIST* expression in sperm cells is no longer detectable after fertilization.

*XIST* expression is first detected at the 4-cell stage in mouse embryos and the 8-cell stage in human and rabbit embryos [9,36,40,41]. In this study we show that in bovine embryos expression of *XIST* is upregulated after the 8-cell stage, and that expression is predominantly observed in female embryos. This is in agreement with previous studies in which the expression of *XIST* was detected after the 16–32-cell stage and was higher in female embryos than male embryos at both the morula and the blastocyst stages [42,43]. Recently, presence of *XIST* transcripts has been reported as early as the 2-cell stage in bovine embryos [39]. The levels of expression were not quantified, however, and may have been very low. Indeed, *XIST* transcripts were detected in all examined embryos and cells at the 2-cell stage [39] where one would not expect functional *XIST* levels in male embryos, suggesting that if expressed, the *XIST* levels would be very low at these stages. Notably, the embryonic genome is activated at around the 2–4-cell stage in the mouse, the 4–8-cell stage in the human, and the 8–16-cell stage in rabbit and cattle embryos [44,45], suggesting that mammalian XCI initiation closely follows activation of the embryonic genome.

The expression of other factors involved in XCI was also examined in this study. Higher expression of *HPRT1* was detected at the 8-cell and the morula stages in female embryos compared to male embryos, but no difference in *HPRT1* expression was found between female and male blastocysts, indicating the normalization of *HPRT1* expression between female and male embryos is accomplished soon after the initiation of XCI. However, the expression of genes coding for polycomb complex components and other XCI activators was not significantly different between female and male embryos during early development. More recently, it has been reported that in mouse embryos SPEN is recruited to the X chromosome immediately upon *Xist* upregulation [46]. Nevertheless, we did not detect significant upregulation of *SPEN* expression from morulae to blastocysts in our microarray data, while *XIST* expression was significantly upregulated. This may be due to their multiple functions during development or because of alternative functions that are not conserved between mouse and cattle.

The combination of RNA FISH with immunofluorescence enabled simultaneous localization of *XIST* RNA and H3K27me3 at the single-cell level. This is technically challenging because the reaction conditions of the two procedures conflict in several ways [47]. Here we found cytoskeletal buffer supplemented with Triton X-100 to be optimal for permeabilization. In mouse and human preimplantation embryos, “pinpoint” signals of *XIST* were observed in both female and male cells, and regarded to indicate an early stage of XCI [48,49]. We did not observe pinpoint-like *XIST* accumulation in any of the female or male embryos examined. Even though we observed that the *XIST* cloud was smaller in female morulae than female blastocysts, it was still larger than the pinpoints described, which were around 100 times smaller than a full *XIST* cloud [48]. We therefore hypothesize that the accumulation of *XIST* on the future inactive X is faster during bovine embryo development than in the mouse and human.

Another interesting observation obtained by RNA FISH was the accumulation of *XIST* on both X chromosomes during embryo development. This phenomenon has also been observed in human and rabbit embryos [9,48], but it is still poorly understood. In our study, the existence of two *XIST* spots was detected in the majority of female embryos we examined, but in a relatively small percentage of cells. Possibly, cells with two XIST spots were mixoploid and contained more than two X chromosomes. In particular, diploid-triploid mixoploidy is common in bovine blastocysts, with mixoploidy presented in around 10% of the cells [50]. Indeed, two XIST spots were also detected in HEK293 cells that contained supernumerary X chromosomes, and we never observed cells with two *XIST* spots in differentiated oviduct epithelial cells.

Alternatively, two *XIST* spots in female cells represent a transient inactive X-chromosome state when the cells exit from naive pluripotency [51]. However, we did not observe any difference in the percentages of cells with two spots in CDX2-positive trophectoderm and CDX2-negative presumed pluripotent cells. Only in day 8 blastocysts, but not in day 7 or day 9 blastocysts, did we observe a slightly higher percentage of cells with two XIST spots in NANOG positive compared to NANOG negative cells.

In mice, the two active X chromosome state is regarded as a hallmark of naïve pluripotency in female ES cells and embryonic cells [52,53]. However, two active X chromosomes are considered unstable in human ES cells, and *XIST* accumulation on X chromosomes was detected in ICM cells of human and rabbit embryos [9,15], indicating that this hallmark may not be conserved across mammals. Here, we also detected *XIST* expression in bovine ICM cells using both qRT-PCR and RNA FISH combined with immunofluorescence. Overall, fewer *XIST* spots were observed in ICM cells than trophectoderm cells. The patterns of *XIST* spot numbers we observed were very similar between CDX negative (presumed epiblast and hypoblast) and NANOG positive (presumed epiblast) cells, which suggests the levels of XCI in epiblast and hypoblast cells are similar in bovine embryos. Indeed, we have previously observed that ICM cells can switch from NANOG positive epiblast precursors to GATA6 positive hypoblast cells, and vice-versa, even in day 8 blastocysts, indicating a large degree of fate flexibility [54]. Besides, like human ES cells, ES cells derived from bovine ICM cells display epigenetic features of the primed rather than the naïve pluripotent state [19]. Taking all together, we suggest the reason why XCI was also initiated in ICM cells in cattle is that those cells are already in primed rather than naïve pluripotent state.

In mouse embryos, reduced expression of X-linked genes was observed after *XIST* accumulation on the X chromosome during preimplantation development [55]. However, in bovine embryos, we found *XIST* accumulation did not lead to globally reduced expression of X-linked genes, similarly to what has been reported for human embryos [9]. In agreement with our findings, microarray and RNA-seq analyses in bovine blastocysts demonstrated enhanced expression of X-chromosome-linked genes in intact female compared with male blastocysts, which indicates that dosage compensation had not initiated at this stage [21,56]. Interestingly, we did observe a significant increase of intense H3K27me3 spots within nuclei from the morula to the blastocyst stage, indicating that this histone modification also did not lead to global downregulation of X-linked genes. Chromatin immunoprecipitation could help to identify the genes on the X chromosome that are specifically linked with H3K27me3.

## 5. Conclusions

We observed the onset of XCI at around the morula stage of bovine embryo development. At the blastocyst stage, ICM cells demonstrate a lower incidence of XCI than TE cells. Moreover, *XIST* accumulation does not lead to global downregulation of X-linked genes. Our data confirmed diverse patterns of XCI initiation exist in different mammalian species during early development.

## Supplementary Materials

The following are available online at https://www.mdpi.com/2073-4409/9/4/1016/s1.
