# Supplementary material for: Initiation of X Chromosome Inactivation during Bovine Embryo Development
Source: Cells. 2020 Apr 19;9(4):1016. doi: 10.3390/cells9041016 (PMC7226380; doi:10.3390/cells9041016)
Supplement: Supplementary file 1 [file cells-09-01016-s001.pdf]

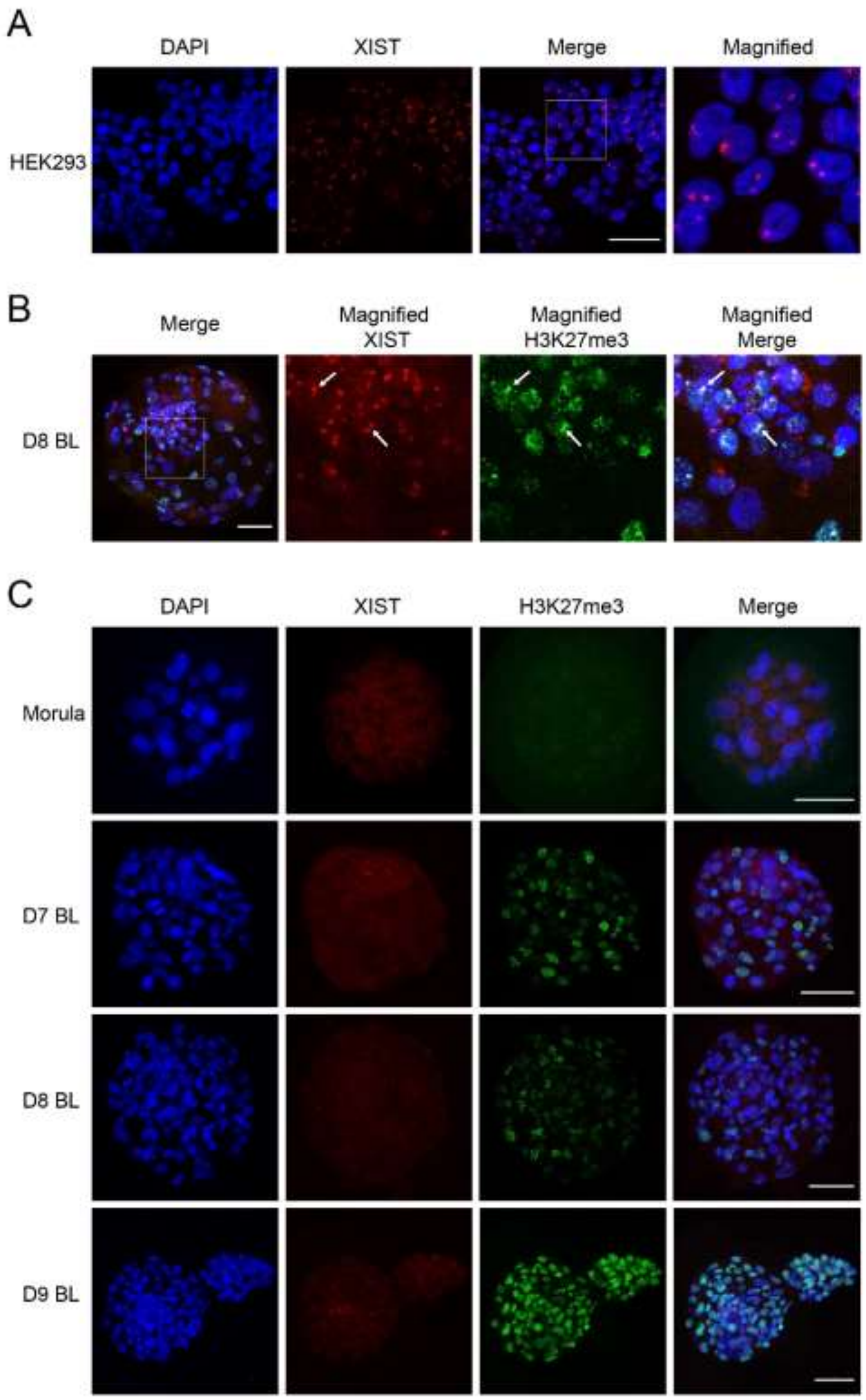

Supplementary Figure 1. XIST RNA FISH in HEK293 cells, female and male blastocysts. HEK2983 cells exhibit one or no XIST spots within nuclei. White box indicates the magnified part presented on the right (A). Day 8 female blastocyst stained for XIST and H3K27me3. White square indicates the magnified parts on the right, showing XIST, H3K27me3 and merged staining. XIST and H3K27me3 colocalization area are indicated (arrows) (B). Absence of XIST and H3K27me3 spots in cells from male morulae to male day 7, 8 and 9 blastocysts (C). Scale bar = 50µm. BL=blastocyst. D=day.

Supplementary Table1. List of primers used for quantitative RT-PCR and embryo sexing. Sense and anti-sense sequences are indicated by s and as respectively. Ta = annealing temperature.

Gene	NCBI	Sequence (5'-3')	Sense	Ta (°C)
CDX2	NM_001206299.1	AACCTGTGCGAGTGGA	s	60
		GCGACTGTAGTGAAACTCC	as	
DDX3Y	NM_001172595.1	GGACGTGTAGGAAACCTTGG	s	63
		GCCAGAACTGCTACTTTGTCG	as	
EED	NM_001040494.2	ATAGCAACCCGGACCTCTCT	s	61
		TCCAGGTGCATTTGGCGTAT	as	
EZH2	NM_001193024.1	GGGCACAGCAGAAGAGCTAA	s	61
		AAGTGTTGGGTGTTGCATGA	as	
GAPDH	NM_001034034.2	AGGCCATCACCATCTTCCAG	s	61
		GGCGTGGACAGTGGTCATAA	as	
HNRNPK	NM_001034562.1	TTGTTTAATCCGCTGACCAC	s	58
		GCAGATGGCTTATGAACCACA	as	
HNRNPU	NM_001076920.2	GTGACCACCCAATCCGAACT	s	61
		GGAGGAAGGGCATCTTACGG	as	
HPRT1	NM_001034035.2	TGCTGAGGATTTGGAGAAGG	s	58
		CAACAGGTCGGCAAAGAACT	as	
JPX	KU050194.1	GGAATGTCCAGAAACGCGGAT	s	61
		GGGCAGGTGAGGTTCAAGGA	as	
OCT4	NM_174580.3	TAGCCACATCGCCCAGCAGC	s	60
		GAAAGGAGACCCAGCAGCCTCA	as	
RING1	NM_001105051.1	GCCGCCTCACACGCT	s	61
		GCCCTACCTGCCGAAAGAA	as	
SDHA	NM_174178.2	GCAGAACCTGATGCTTTGTG	s	64
		CGTAGGAGAGCGTGTGCTT	as	
SOX2	NM_001105463	CCATGCAGGTTGACATCGT	s	60
		ACACAACTACGGAAACTAAAAGTGG	as	
USP9Y	NM_001145509.1	GCCAGATGACCAAGAAGCCCCA	s	63
		GGACTGTAAGGCCTAATAGCCTGGT	as	
XIST	AF104906.5	AGCATTGCTTAGCATGGCTC	s	61
		TGGCTGTGACCGATTCTACC	as	
YWHAZ	XM_025001429.1	GCATCCCACAGACTATTTCC	s	56
		GCAAAGACAATGACAGACCA	as	
ZRSR2Y	GQ426330.1	GTCAGTTGCAACCTGGAACC	s	63
		GCCATATTCCATTGGGTCAC	as	

Supplementary Table 2. List of oligonucleotide sequences used for XIST RNA FISH.

No.	Sequence (5'-3')	No.	Sequence (5'-3')
XIST-1	CAGTGGTAGTGAATCTTTCC	*XIST*-25	TCTTATCACTTGGGAGCATT
XIST-2	CTTTGCACTACTTCAGGGAA	*XIST*-26	GTCTGTCTCAACCTAAGTTT
XIST-3	TCAAGCCAACAAGGGGAACG	*XIST*-27	GGAGAAGGGAATGACAACCC
XIST-4	AGGGTAGCCAGTATCAGAAA	*XIST*-28	AGTTCAGTTTCTGGGTCAAG
XIST-5	AAAGAGACACTGGGGCACAC	*XIST*-29	ATACTGCCTTTCTCATAGTG
XIST-6	GCACACACACAAACACGCAT	*XIST*-30	ACCCATTCATTTACCAAGTA
XIST-7	TTTGATAGCTGACACTCTGC	*XIST*-31	GTGCCTATAACAGTGAGTTC
XIST-8	GTTTTGATCATCTCACAGGG	*XIST*-32	TCATTCAGCAGATATGGCTT
XIST-9	GAGACTTGAGTCTTCTATCC	*XIST*-33	GCAATGAATTTTTGCATCCC
XIST-10	CTGGTATCTCTGTCACGAAC	*XIST*-34	TCCTAGAGCATACACAGTTT
XIST-11	TCTTGGAATTTCCTTTGGTC	*XIST*-35	GTCTGGATTTTATCTTTCCA
XIST-12	GCAATAGAAACAGCTGGGGT	*XIST*-36	CTTATGCTCAGGACCTAGAA
XIST-13	CTGAACCTGATACTTTCCTT	*XIST*-37	TCCAAGGCAAGTCAATAGGT
XIST-14	TAATTCTTCTCATTGGCCTG	*XIST*-38	ATCTTTTTTGTTGTCTTCCT
XIST-15	CAATCCTCCTTCATAACCAA	*XIST*-39	AATCTCTTCATTCTCCTCAG
XIST-16	CTATGAGCAGGGAGTTCATG	*XIST*-40	TCATTCCATCTCAGTATCTT
XIST-17	TTACCACATTATTAGGCCAC	*XIST*-41	ATCACCTTCTTCAATGCTTG
XIST-18	AATTGCCATCTGATCCTTTA	*XIST*-42	CCTCTTCTCTTATATACCAG
XIST-19	GAGAGAGACCATATGGCTTG	*XIST*-43	GGGTTAGCTAGAGAACCTAG
XIST-20	CCTTAATGCATACAGGGACA	*XIST*-44	TTAAGTTCTCTGGGAAGGCA
XIST-21	CCAGAGCCACAGAAACACAA	*XIST*-45	CCATTTTTCATGAGGGATGG
XIST-22	CAGACACGACTTAGCGACTG	*XIST*-46	TTTGGGAATCAGTTGCTTCT
XIST-23	TTGTGTTTGCAGTATGCTTT	*XIST*-47	AGGTTGGTACCAATGAGACT
XIST-24	CATATAGCCCATTGGTATTT	*XIST*-48	TCCATCTCCAAACTTGCTAA

Supplementary Table 3. Detection of genes, as indicated, on genomic DNA in blastocysts generated by X- and Y-sorted sperm.

	DDX3Y (+), USP9Y (+), ZRSR2Y (+)		DDX3Y (-), USP9Y (-), ZRSR2Y (-)		GAPDH (+)		Total number of blastocysts
	(N)	%	(N)	%	(N)	%	
Unsorted sperm	8	53.3	7	46.7	15	93.8	16
X-sorted sperm	0	0	12	100	12	100	12
Y-sorted sperm	11	91.7	1	8.3	12	100	12

Supplementary Table 4. Differentially expressed genes on the X-chromosome between blastocyst and morula. All genes differentially expressed (1.5-fold; p<0.05) on the X-chromosome between blastocyst and morula are listed. FC = fold change. Positive FC values represent higher expression in blastocyst and negative values represent lower expression in blastocyst, compare with morula.

Gene Symbol (NCBI)	FC	P-value		Gene Symbol (NCBI)	FC	P-value
PAGE4	103,89	0,00E+00		RNF128	-11,28	1,02E-03
unknown	8,93	0,00E+00		BC149657	-10,66	1,46E-04
SH3BGRL	7,41	0,00E+00		TKTL1	-7,76	2,51E-04
AIFM1	6,31	6,84E-06		PRPS1	-7,35	3,77E-04
TCEAL4	6,04	8,47E-06		FOXO4	-6,13	2,51E-05
CASK	5,62	0,00E+00		SRPX	-5,94	4,21E-06
unknown	5,47	0,00E+00		MGC148328	-5,89	4,57E-03
ATP6AP1	5,04	2,10E-04		NUDT10	-5,67	4,64E-07
CITED1	4,59	2,48E-07		MOSPD1	-5,34	3,88E-06
MTMR1	4,56	8,08E-07		DT836038	-5,33	1,23E-03
MAGED2	4,29	3,89E-03		SSX5	-4,82	6,33E-07
IL13RA1	4,24	3,79E-05		DY197030	-4,74	6,33E-07
GPR34	4,15	6,33E-07		NUDT11	-4,70	6,17E-04
TMEM47	3,98	8,72E-04		GK	-4,64	2,48E-07
DV781488	3,88	4,42E-06		FHL1	-4,37	2,52E-06
EH181383	3,87	3,60E-06		unknown	-4,27	4,64E-07
LANCL3	3,85	8,46E-05		HPRT1	-4,00	1,13E-04
PGRMC1	3,66	1,97E-06		TIMM8A	-4,00	2,16E-03
unknown	3,58	9,95E-06		ENSBTAT00000021646	-3,68	2,60E-06
ENSBTAT00000011172	3,56	2,10E-05		RNF113A	-3,66	4,72E-06
TCEAL1	3,49	1,45E-04		MAGEB16	-3,58	2,60E-06
MOSPD2	3,43	4,32E-06		CUL4B	-3,56	3,59E-04
GRPR	3,42	2,02E-04		unknown	-3,39	2,75E-05
EBP	3,30	1,38E-05		LOC786770	-3,23	9,90E-05
LOC783604	3,30	2,66E-05		CK730304	-3,15	1,93E-05
LAMP2 tv 1	3,21	3,47E-02		OGT	-3,12	2,66E-05
PRAF2	3,02	1,72E-05		CT47B1	-3,06	1,14E-05
AP1S2	3,00	3,52E-03		BMP15	-3,05	1,17E-04
MBTPS2	2,95	5,04E-05		ENSBTAT00000024713	-2,95	2,00E-05
PJA1	2,93	2,00E-05		ENSBTAT00000021597	-2,86	3,50E-05
XIST	2,86	1,39E-04		HMGB3	-2,84	2,26E-04
DV910827	2,74	1,15E-03		RBM10	-2,67	9,52E-05
MTCP1NB	2,72	6,63E-05		VBP1	-2,66	2,87E-02
LAMP2 tv 2	2,72	1,92E-04		ENSBTAT00000026055	-2,54	1,31E-02
ENSBTAT00000000756	2,68	8,62E-04		RPS6KA3	-2,51	1,78E-03
unknown	2,66	4,50E-03		BC142046	-2,46	9,68E-05
NSDHL	2,66	8,27E-03		TSR2	-2,45	3,44E-03
ARMCX3	2,63	2,17E-02		CCDC160	-2,40	4,10E-04
HDHD1	2,61	1,87E-04		BC110239	-2,36	3,99E-02

CTPS2	2,56	9,92E-05		PLS3	-2,30	1,11E-03
MOSPD2	2,52	1,05E-04		unknown	-2,30	5,60E-04
EH196820	2,47	3,44E-04		NKRF	-2,29	3,22E-04
ANOS1	2,45	3,86E-04		unknown	-2,28	2,05E-04
NHSL2	2,45	3,41E-03		unknown	-2,28	7,67E-04
MST4	2,43	4,51E-03		unknown	-2,27	6,23E-04
RAP2C	2,42	2,16E-04		MSL3	-2,26	7,40E-03
COL4A5	2,38	1,14E-02		ENSBTAT00000063298	-2,26	2,00E-03
GPR173	2,37	2,11E-02		SPIN2B	-2,23	2,46E-04
EE376747	2,34	2,48E-04		ENSBTAT00000015084	-2,22	2,46E-02
GPR34	2,30	1,94E-04		unknown	-2,20	1,05E-03
unknown	2,30	1,75E-04		EIF1AX	-2,20	3,66E-02
DYNLT3	2,24	2,38E-04		CB456663	-2,19	3,22E-03
C1GALT1C1	2,21	3,16E-04		LOC100336029	-2,15	2,34E-02
DT837923	2,19	2,92E-03		DT851156	-2,15	3,31E-03
PLP2	2,19	4,06E-04		BC151770	-2,14	9,22E-03
KCNE1L	2,19	2,80E-03		DDX3X	-2,14	3,95E-04
unknown	2,18	8,03E-04		IL2RG	-2,11	7,91E-04
ATP6AP2	2,15	2,47E-02		TRAPPC2	-2,10	5,30E-03
MPP1	2,15	2,87E-03		unknown	-2,10	4,68E-04
CO886779	2,14	4,10E-04		BC149670	-2,09	1,36E-02
LOC615809	2,13	6,54E-04		KIF4A	-2,09	3,31E-03
LOC508820	2,13	1,04E-02		BEX2	-2,07	3,15E-03
RAB9A	2,07	5,24E-04		NXF2	-2,06	8,31E-04
unknown	2,07	2,12E-03		MAOA	-2,05	7,85E-04
ARMCX2	2,04	2,85E-03		unknown	-2,05	7,02E-04
AB098746	2,03	2,87E-02		ENSBTAT00000023863	-2,04	6,56E-04
CFP	2,01	7,68E-04		ENSBTAT00000013340	-2,04	3,88E-02
FTL	1,99	2,26E-03		ZFY	-2,03	1,45E-02
SH3KBP1	1,96	2,32E-03		MGC134232	-2,03	6,80E-04
ZNF75D	1,92	1,15E-03		PRRG1	-2,03	4,06E-03
ZC4H2	1,91	1,67E-03		unknown	-2,00	5,15E-03
ZMYM3	1,91	2,79E-03		CXHXorf26	-1,98	5,48E-03
LOC100849861	1,91	5,26E-03		BC134574	-1,95	1,99E-03
UTP14A	1,91	6,30E-03		CXHXorf65	-1,94	2,29E-03
unknown	1,89	1,59E-03		RAN	-1,93	3,45E-03
FLNA	1,87	1,92E-03		MID1	-1,93	3,01E-03
ENSBTAT00000021407	1,85	2,82E-03		TMEM164	-1,90	1,29E-03
TSPAN6	1,84	1,37E-02		RBBP7	-1,90	2,58E-02
TMLHE	1,83	1,89E-03		WDR45	-1,89	1,47E-03
APOOL	1,83	2,06E-03		NXF3	-1,89	2,64E-03
ENSBTAT00000019400	1,75	7,94E-03		NONO	-1,87	3,63E-02
unknown	1,74	3,79E-03		HNRNPF	-1,87	2,57E-02
ZNF75D	1,73	1,35E-02		TMEM185A	-1,86	2,23E-03
ABCD1	1,73	3,72E-03		NXT2	-1,86	1,64E-03

SAGE1	1,73	6,14E-03		ENSBTAT00000027211	-1,85	4,50E-03
ENSBTAT00000006967	1,72	4,71E-03		STAG2	-1,85	1,99E-03
BC142193	1,72	1,45E-02		GNL3L	-1,84	1,88E-03
BC142083	1,72	4,58E-03		GLA	-1,83	2,24E-03
ASB11	1,72	4,01E-03		LOC100126054	-1,83	2,66E-03
BE749580	1,71	6,42E-03		REPS2	-1,82	8,14E-03
ABCB7	1,70	8,41E-03		USP9Y	-1,81	2,87E-03
SSR4	1,70	4,73E-03		MED12	-1,81	1,25E-02
TFE3	1,68	5,77E-03		SLC25A14	-1,80	3,66E-03
unknown	1,68	3,55E-02		RENBP	-1,79	2,10E-02
SLC9A6	1,64	1,06E-02		CETN2	-1,79	2,53E-03
RGN	1,64	4,31E-02		ENSBTAT00000004701	-1,78	2,93E-03
DLG3	1,63	1,68E-02		unknown	-1,78	2,08E-02
BC134601	1,60	2,66E-02		DKC1	-1,76	3,07E-03
PIM2	1,59	1,04E-02		ENSBTAT00000021068	-1,75	4,91E-03
PSMD10	1,59	9,31E-03		BC108185	-1,75	6,70E-03
unknow	1,58	1,14E-02		ENSBTAT00000048938	-1,75	3,50E-02
GLRA4	1,57	1,99E-02		GNL3L	-1,75	4,40E-03
unknown	1,56	1,19E-02		ENSBTAT00000028200	-1,74	4,78E-03
ENSBTAT00000029281	1,56	1,26E-02		TM9SF2	-1,73	4,06E-02
ENSBTAT00000015650	1,56	1,29E-02		HCCS	-1,73	4,32E-03
DV879767	1,54	1,41E-02		EIF1AY	-1,72	1,53E-02
DOCK11	1,53	4,93E-02		BHR6A	-1,70	1,41E-02
FAM127C	1,53	2,22E-02		CK776385	-1,68	1,44E-02
unknown	1,53	2,28E-02		unknown	-1,67	4,51E-02
TCEAL8	1,52	2,87E-02		PRDX4	-1,66	6,80E-03
				UPF3B	-1,66	1,19E-02
				RBMX2	-1,66	2,15E-02
				PFKFB1	-1,63	8,64E-03
				BC108149	-1,62	1,58E-02
				DN535421	-1,61	8,39E-03
				CXHXorf56	-1,60	3,53E-02
				MGC152340	-1,60	8,72E-03
				SUV39H1	-1,60	1,61E-02
				unknown	-1,59	1,14E-02
				CSNK1B	-1,59	1,73E-02
				ENSBTAT00000064722	-1,59	2,63E-02
				BCOR	-1,55	1,27E-02
				HEPH	-1,53	2,01E-02
				BC120247	-1,51	2,39E-02
				ENSBTAT00000046182	-1,51	2,18E-02
				LOC789689	-1,51	2,82E-02
				unknown	-1,50	3,22E-02

Supplementary Table 5. Differentially expressed genes on the X-chromosome between inner cell mass and trophectoderm. All genes differentially expressed (1.5-fold; p<0.05) on the X-chromosome between inner cell mass and trophectoderm are listed. FC = fold change. Positive FC values represent higher expression in inner cell mass and negative values represent lower expression in inner cell mass, compared with trophectoderm.

Gene Symbol (NCBI)	FC	P-value		Gene Symbol (NCBI)	FC	P-value
GPC4	7,77	0,00E+00		NSDHL	-2,24	2,93E-03
TKTL1	6,43	1,48E-05		PAGE4	-2,23	4,67E-03
BTK	5,61	0,00E+00		unknown	-2,23	5,70E-05
SH3BGRL	4,47	0,00E+00		TSPAN7	-2,11	1,07E-04
LOC100139405	3,51	1,25E-02		ENSBTAT00000054636	-2,01	1,79E-04
L1CAM	3,50	0,00E+00		unknown	-1,91	3,43E-02
BEX2	3,24	1,16E-03		MBTPS2	-1,88	3,79E-03
AP1S2	3,19	2,45E-03		LOC523454	-1,86	6,77E-03
BC149657	3,16	0,00E+00		ATP6AP1	-1,82	7,39E-04
BCOR	2,87	1,55E-03		ENSBTAT00000000756	-1,73	3,58E-03
IL13RA1	2,66	2,52E-03		PSMD10	-1,67	1,07E-03
SLC7A3	2,45	5,30E-05		CD99	-1,67	1,94E-02
unknown	2,43	1,08E-05		MOSPD2	-1,67	5,64E-03
ENSBTAT00000048938	2,33	3,67E-04		LOC787476	-1,65	4,88E-02
FOXO4	2,32	1,04E-04		SAT1	-1,62	2,67E-02
ARMCX2	2,27	1,68E-03		CO886779	-1,60	9,06E-03
PLS3	2,21	1,11E-04		EBP	-1,58	2,98E-03
PIM2	2,12	2,29E-04		COL4A5	-1,58	3,56E-02
STAG2	1,94	3,85E-04		ENSBTAT00000019400	-1,53	3,75E-03
ARMCX1	1,93	3,86E-02		ASB11	-1,53	1,47E-02
unknown	1,89	2,37E-04		RAB9A	-1,52	2,09E-02
ENSBTAT00000002444	1,85	4,00E-04		WDR44	-1,50	3,07E-02
HSD17B10	1,81	3,07E-02				
MID1	1,77	3,81E-03				
ENSBTAT00000038974	1,76	5,99E-03				
unknown	1,72	2,84E-03				
ZMYM3	1,70	1,72E-03				
ARX	1,69	1,41E-03				
TSR2	1,68	2,59E-03				
LOC538872	1,68	5,53E-03				
unknown	1,63	6,29E-03				
CB456663	1,61	2,02E-03				
ENSBTAT00000017619	1,61	1,97E-02				
RRAGB	1,61	4,23E-02				
BP107448	1,58	3,82E-02				
ENSBTAT00000011843	1,54	3,47E-03				
FTL	1,52	4,37E-03				
